# Awake craniotomy does not lead to increased psychological complaints

**DOI:** 10.1007/s00701-023-05615-5

**Published:** 2023-05-24

**Authors:** I. M. C. Huenges Wajer, J. Kal, P. A. Robe, M. J. E. van Zandvoort, C. Ruis

**Affiliations:** 1 Department of Neurology and Neurosurgery, University Medical Center Utrecht, Utrecht, The Netherlands; 2 Department of Experimental Psychology, Helmholtz Institute, Utrecht University, Utrecht, The Netherlands

**Keywords:** Awake craniotomy, Brain tumours, Psychological complaints, Anxiety, Depression, PTSD

## Abstract

**Background:**

Patients with brain tumours are increasingly treated by using the awake craniotomy technique. Some patients may experience anxiety when subjected to brain surgery while being fully conscious. However, there has been only limited research into the extent to which such surgeries actually result in anxiety or other psychological complaints. Previous research suggests that undergoing awake craniotomy surgery does not lead to psychological complaints, and that post-traumatic stress disorders (PTSD) are uncommon following this type of surgery. It must be noted, however, that many of these studies used small random samples.

**Method:**

In the current study, 62 adult patients completed questionnaires to identify the degree to which they experienced anxiety, depressive and post-traumatic stress complaints following awake craniotomy using an awake-awake-awake procedure. All patients were cognitively monitored and received coaching by a clinical neuropsychologist during the surgery.

**Results:**

In our sample, 21% of the patients reported pre-operative anxiety. Four weeks after surgery, 19% of the patients reported such complaints, and 24% of the patients reported anxiety complaints after 3 months. Depressive complaints were present in 17% (pre-operative), 15% (4 weeks post-operative) and 24% (3 months post-operative) of the patients. Although there were some intra-individual changes (improvement or deterioration) in the psychological complaints over time, on group-level postoperative levels of psychological complaints were not increased relative to the preoperative level of complaints. The severity of post-operative PTSD-related complaints were rarely suggestive of a PTSD. Moreover, these complaints were seldom attributed to the surgery itself, but appeared to be more related to the discovery of the tumour and the postoperative neuropathological diagnosis.

**Conclusions:**

The results of the present study do not indicate that undergoing awake craniotomy is associated with increased psychological complaints. Nevertheless, psychological complaints may well exist as a result of other factors. Consequently, monitoring the patient’s mental wellbeing and offering psychological support where necessary remain important.

**Supplementary information:**

The online version contains supplementary material available at 10.1007/s00701-023-05615-5.

## Introduction

The treatment of brain tumours increasingly consists of surgery in which the patient is fully conscious [5]. The choice for an awake craniotomy is based on whether the tumour is located in an eloquent area of the brain: a part of the brain that is crucial for (part of) neurological functions. By stimulating the brain during surgery and simultaneously conducting neuropsychological tests, the loss of a particular cognitive function can be linked to the stimulated area of the brain [8, 26]. This allows for the localisation of functions in order to retain them as much as possible during the tumour resection. During an awake craniotomy, much more of the tumour tissue can be removed compared to when the patient is operated under general anaesthesia, because functional areas can be determined more accurately. Research has shown that patients develop less severe neurological deficits after an awake craniotomy than patients who are not awake during surgery [6]. Moreover, awake craniotomy reduces the duration of the hospital admission [9].

In an awake craniotomy, many patients are operated using an ‘asleep-awake-asleep’ method. This entails that the patient is sedated when the skull is opened and closed. In between, the patient is awakened to allow for the administration of neuropsychological tests. This method is described as a safe, effective surgical technique that is tolerated well by patients [18]. During an awake craniotomy, different anaesthesia techniques are used to ensure patient safety but also to control anxiety and pain during the procedure [2]. However, the use of anaesthesia at the start and end of the surgery leads to certain risks, such as nausea, hypoventilation, and respiratory tract obstructions. Patients may also be confused and agitated when awakened in the operating room, and the residual effects of sedation may negatively affect their alertness, cooperation, and performance during the intra-operative tests [13, 14, 20]. To minimize these risks, neurosurgeons may choose for an ‘awake-awake-awake’ procedure instead, in which the patient is conscious throughout the surgery. An alternative procedure involves the patient being sedated after the intra-operative tests (‘awake-awake-asleep’). The awake-awake-awake procedure therefore presents several benefits. To be able to apply this method, however, it is important to know how patients experience the procedure. Undergoing brain surgery is already an event with a major impact for the patient and his/her loved ones, and the impact may even be greater when the patient is fully conscious from the start of the surgery to the end. It is plausible to consider that this type of surgery may lead to an increased level of anxiety or stress. It is known that patients may suffer from symptoms of a post-traumatic stress disorder (PTSD) following other surgical treatments, such as lung surgeries, abdominal surgeries, and heart surgeries [12]. It is possible that the same applies to awake craniotomy. There is however limited research on the psychological effects of this type of surgery.

Not only the surgery, but also the diagnosis of the brain tumour may have a major impact on patients and their loved ones. Being confronted with a life-changing diagnosis may be experienced as a traumatic event [25]. A meta-analysis has shown that 23% of patients suffer from PTSD in the first year following a stroke or transient ischemic attack (TIA), with a reduction to 11% after the first year [11]. Although brain tumours do not always cause acute symptoms at the moment they are discovered, it is conceivable that the discovery of a tumour may be a traumatic event. Another factor that may cause anxiety is the potential future deterioration in functioning. According to the Enduring Somatic Threat (EST) model described by Edmondson (2014), PTSD resulting from medical events differs in many aspects from PTSD due to other causes [10]. Although many traumatic events result in extreme anxiety for a past event, such as an accident or violence, many medical conditions result in anxiety due to a persistent threat. The threat posed by medical conditions is also localised within one’s own body, where other causes of PTSD are related to an external threat factor. According to Edmondson, these factors make psychological trauma in medical conditions both unique and complex.

Over the past decade, some research has been conducted on PTSD following awake craniotomy for brain tumours. This research has shown that 1 out of 8 patients experience PTSD symptoms, such as nightmares, following an awake-awake-awake procedure [19]. However, this research only involved a small random sample of 16 patients. A review of Milian et al. (2014) shows that none of the patients who had undergone an awake craniotomy actually met the criteria for PTSD over the short or long term [20]. This applies to both the asleep-awake-asleep and the awake-awake-awake procedure.

The finding that PTSD and PTSD symptoms are rare after awake craniotomy does not mean that patients do not experience psychological complaints at all after this type of surgery. It is therefore relevant to identify the extent to which this patient group experiences psychological complaints, and whether the post-operative level of these complaints differs from the pre-operative level. A more recent study considered the presence and course of psychological complaints following awake craniotomy (awake-awake-asleep). This study showed that the level of psychological complaints did not increase after surgery. Here, too, one should note that the study involved a small random sample of only 20 patients [15].

In the current study, the level and course of psychological complaints after awake-awake-awake craniotomy is studied by identifying the level of anxiety, depressive complaints, and post-traumatic stress symptoms in patients with a brain tumour, both before and after surgery. The aim of this study is also to investigate the degree to which patients attribute any post-traumatic stress complaints to the discovery of the tumour, the surgery itself, and the postoperative neuropathological diagnosis.

## Methods and materials

### Participants

All participants in this study are patients with a brain tumour who have been treated with awake craniotomy (awake-awake-awake procedure) at the University Medical Centre Utrecht (UMCU) between October 2014 and July 2016. All patients were over the age of 18 years. All patients were able to speak Dutch and possessed adequate language comprehension, so that comprehension problems could not influence the reliability of the questionnaires. Moreover, only patients who had brain surgery for the first time were included, to prevent previous experiences with neurosurgery from playing a role in the emotional experience of the procedure.

During the period of the study, 102 patients underwent an awake craniotomy. Of these, 16 patients did not meet the inclusion criteria: 11 patients had already undergone neurosurgery before (awake or asleep), 4 patients suffered from reduced language comprehension, and 1 patient was not sufficiently proficient in Dutch.

Of the 86 patients who met the inclusion criteria, 1 patient did not participate in the study due to a very poor medical condition. 3 patients died during the study period, and 4 patients did not give permission for the use of their data. Another 16 participating patients were excluded over the course of the study period due to incomplete data at 4 weeks (T1) and 3 months (T2) following the surgery. This brings the final number of participating patients to 62 of which 41 (66%) are male and 21 (34%) are women, presenting with oligodendrogliomas (25%), astrocytomas (21%), glioblastomas IDH WT (48%), supratentorial ependymoma (2%) or metastases (2%) (Table 1).

**Table 1:** Demographic data and brain tumour characteristics (*N* = 62)

Age on day of surgery (in years)	M = 54 (SD = 13.1) Range 21–78
Sex	
- Male	41 (66%)
- Female	21 (34%)
Tumour type	
* Gliomas*	
Oligodendroglioma	
• IDH mutant, 1p19q codel, CNS WHO grade 2	5 (8%)
• IDH mutant, 1p19q codel, CNS WHO grade 3	9 (15%)
• NEC, CNS WHO grade 3	1 (2%)
Astrocytoma	
• IDH mutant, CNS WHO grade 2	7 (11%)
• IDH mutant, CNS WHO grade 3	3 (5%)
• IDH mutant, CNS WHO grade 4	2 (3%)
• IDH WT, CNS WHO grade 2	1 (2%)
Glioblastoma IDH WT	30 (48%)
* Ependymal tumours*	
Supratentorial Ependymoma, NOS, CNS WHO grade 3	2 (3%)
* Metastases*	2 (3%)

Of the total study population, 61.3% filled in the questionnaires on psychological complaints at both post-operative measurements. Of the remaining 38.7%, data are available for one of the two post-operative measurements.

### Questionnaires

To measure anxiety and depressive complaints, the Dutch version of the Hospital Anxiety and Depression Scales (HADS) was used [23]. This self-evaluation questionnaire consists of 14 items that must be answered on a 4-point Likert scale. The questionnaire consists of two sub-scales with a maximum score of 21. Seven items measure anxiety complaints, and seven items measure depressive symptoms. In this study, the two sub-scales are used separately, whereby a score of ≥ 8 is considered to be indicative of anxiety or depression [23]. Based on the SD of the normative data of the anxiety and the depression scale of the HADS [23], for each scale a change of more than ± 3 points between two measurements is considered as a deterioration or improvement on the HADS scale.

To measure PTSD complaints, the Dutch version of the Self-Rating Inventory for PTSD (SRIP) was used [16]. This questionnaire measures the PTSD complaints experienced over the past four weeks and is based on the criteria described in the Diagnostic and Statistical Manual of Mental Disorders (DSM-IV-TR) [1]. The DSM-5 also lists the presence of PTSD complaints for at least 4 weeks as one of the criteria for the PTSD diagnosis. This self-evaluation inventory consists of 22 items that must be answered on a 4-point Likert scale. The questionnaire has three sub-scales: recurrence, avoidance, and hyperarousal. A total score of ≥ 52 is suggestive of PTSD.

To determine which specific events are related to potential PTSD complaints, a visual analogue scale (VAS) was used for three factors: the discovery of the tumour, the awake surgery, and the post-operative neuropathological diagnosis.

### Procedure

All patients were informed about what the surgical procedure (pre, peri and post) entails in depth by the neurosurgeon, the nurse practitioner and the clinical neuropsychologist. In addition to this information, all patients were subjected to an extensive neuropsychological assessment to identify their cognitive functioning between one and five days prior to the surgery. At the end of this assessment, the HADS was administered to measure anxiety and depressive complaints.

During the surgery, local anaesthesia was used, and all patients were fully conscious (for more technical details see *Neurosurgical Procedure*). Their cognitive functions were monitored by the clinical neuropsychologist. The clinical neuropsychologist also monitored the patient’s psychological wellbeing and provided coaching when needed.

The patients received the HADS again and also the SRIP by mail, with the request to complete both questionnaires and return it at an interval of four weeks after the surgery and three months after the surgery. The measurement after four weeks is considered to be an inventory of psychological complaints in the short term after the surgery. The measurement after three months is suitable to identify symptoms over the longer term. In case of increased levels of psychological complaints patients were offered or referred for psychological support.

### Neurosurgical procedure

Patients were operated in a fully awake fashion under local anesthesia, microscope view and ultrasound and neuronavigation guidance. The procedures were performed in a parkbench position, allowing the patients to relax and to face the anesthesist and/or the neuropsychologist in a comfortable fashion. The head was fixed in a Mayfield clamp placed under local anesthesia (using a mix of 5 mg/ml chirocaine 1:1 v:v and 2% lidocaine with adrenaline 1:200,000 (a total of 29-55 cc for the complete procedure) injected at the pin sites of the Mayfield clamp and in a rectangular fashion around the planned skin incision site. After removal of the bone flap using a high-speed Anspach® drill, anesthetics mix-soaked gelatin-foam was applied onto the dura at the level of the meningeal arteries. Patients also received titrated pain sedation and relaxation with remifentanyl and propofol, respectively. Tumours were approached using standard routes, and removed using the CUSA at ultra-low power under constant cortico-subcortical stimulations using a Ojeman® cortical stimulator (50 Hz, 1 ms, 2–4 mAmps, trains of 3 s at the cortical level and continuous stimulations at the subcortical level). None of the patient’s local anesthesia had to be converted to general anaesthesia. Areas of stimulation that provoked deficits in the concomitantly performed neuropsychological tests reliably (i.e., 3 times) were considered as functional for that test. In addition, subcortical stimulations that provoked phosphenes, even only once, were considered as belonging to the optic radiations and respected.

### Statistical analyses

First, the number of patients suffering from anxiety complaints, depressive complaints or PTSD-related complaints before and after the awake craniotomy was identified, based on the cut-off scores (HADS anxiety, HADS depression, and SRIP total score). Subsequently, it was determined whether the level of post-operative anxiety and depressive complaints differed from the pre-operative levels, by means of a paired-samples T-test or, if the scores did not follow a normal distribution, a Wilcoxon Signed Rank Test. In addition, Kendall’s tau correlations were calculated to examine if there were associations between age respectively sex and the level of anxiety, depression and PTSD-related complaints.

Finally, in patients with complaints that were suggestive of PTSD, it was identified to which event they attributed these complaints most, based on the VAS score. Subsequently, for each factor a percentage was calculated that represented the number of patients that attributed their PTSD symptoms mainly to this factor.

IBM SPSS Statistics, version 25 was used for the statistical analyses.

## Results

### Anxiety

**Fig. 1:**
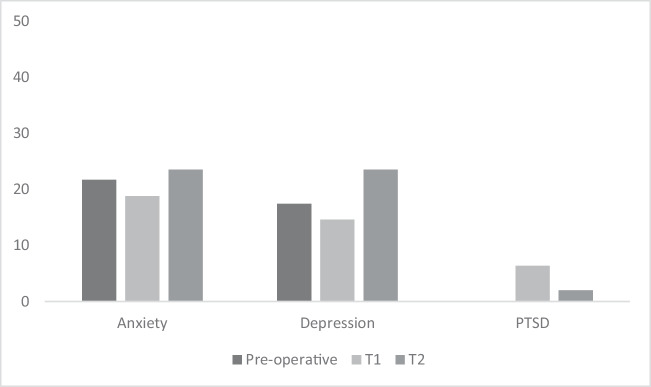
Percentage of patients with a complaint level above the cut-off score at the various measurements in time. The SRIP was only administered at T1 (4 weeks after surgery) and T2 (3 months after surgery)

In the week prior to the surgery (T0), 21.7% of the patients scored above the cut-off score on the anxiety scale. Four weeks after the surgery (T1), this percentage was 18.8%, and three months after the surgery (T2) it was 23.5% (see Fig. 1).

**Fig. 2:**
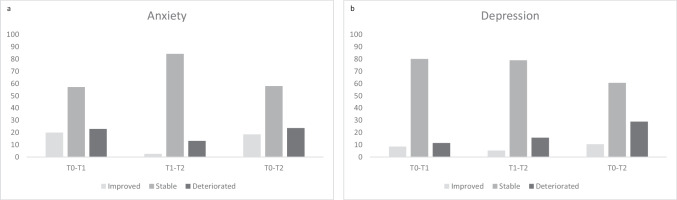
Percentage of patients who showed improvement, stability, or deterioration between different measurements on anxiety (**a**) and depression (**b**) scale of the HADS. T0 = pre-operatively, T1 = 4 weeks after the surgery, T2 = 3 months after surgery

The level of anxiety was only associated with sex in the week prior to the surgery (T0), showing that women reported higher levels of anxiety compared to men (*r*_*τ*_ = *0.321, p* = 0.012). No associations between sex and post-surgery levels of anxiety (T1 & T2) were found. None of the levels of anxiety (T0-T2) was associated with age (see Online Resource 1). In relation to the pre-operative anxiety scores, both four weeks after the surgery (T1) and 3 months after surgery (T2) 7 patients (20%) showed an improvement in anxiety scores. Deterioration in anxiety scores were found in 8 patients (23%) after four weeks (T1) and in 9 patients (24%) 3 months after the surgery (T2). The anxiety scores of the majority of the patients (57% at T1 and 58% at T2) stayed stable over time. More details about the intra-individual changes in anxiety scores between the different measurements are provided in Fig. 2a. To identify whether the level of anxiety complaints had increased after the surgery on group level, the pre-operative level (T0), the scores at T1 and T2 were compared with the pre-operative scores. As the scores on the HADS anxiety scale were not distributed normally, the non-parametric Wilcoxon Signed-Rank Test was used. This test (*n* = 35) shows that the level of anxiety complaints at 4 weeks after the surgery did not differ significantly from the pre-operative level (*Z* = -0.148, *p* = 0.883). The level of anxiety complaints at 3 months after the surgery (*n* = 38) also did not differ from the pre-operative anxiety levels (*Z* = 0.875, *p* = 0.382). No significant difference was observed between the anxiety level at 4 weeks and 3 months after the surgery (*Z* = 1.066, *p* = 0.286) (see Table 2).

**Table 2:** Means and standard deviations for the HADS anxiety and depression scale scores and the SRIP total score at the three measurements and the p-values of the differences between these measurements

	T0	T1	T2	T0-T1 *p*	T0-T2 *p*	T1-T2 *p*
HADS anxiety scale	5.0 (3.7)	4.5 (4.3)	5.0 (4.1)	.883	.382	.286
HADS depression scale	3.9 (3.9)	3.8 (4.0)	4.6 (4.7)	.346	.218	.118
SRIP total score	-	32.7 (8.9)	31.2 (7.7)	-	-	.190

*T0* = *pre-operative; T1* = *4 weeks after surgery; T2* = *3 months after surgery*

### Depression

In the week prior to the surgery (T0), 17.4% of the patients scored above the cut-off score for the HADS depression scale. Four weeks after the surgery (T1), this percentage was 14.6%, and three months after the surgery (T2) it was 23.5% (see Fig. 1).

As the scores on the depression scale were not distributed normally, a non-parametric test was used. In line with complaints of anxiety, complaints of depression were also only associated with sex in the week prior to the surgery (T0). Women reported higher levels of complaints of depression compared to men (*r*_*τ*_ = *0.327, p* = 0.011). No associations between sex and post-surgery levels of depression (T1 & T2) were found. In addition, none of the levels of anxiety (T0-T2) was associated with age (see Online Resource 1). Compared to the pre-operative scores, depression scores had improved in 3 patients (9%) and deteriorated in 4 patients (11%) four weeks post-surgery (T1). Three months after surgery (T2) improvement and deterioration in depression scores were present in respectively 4 (11%) and 11 (29%) of the patients. The depression scores of most of the patients (80% at T1 and 60% at T2) stayed stable over time. More details about the intra-individual changes in depression scores between the different measurements are provided in Fig. 2b. The level of depressive complaints at 4 weeks after the surgery did not differ significantly from the pre-operative level (*Z* = 0.943, *p* = 0.346). Although a higher percentage of patients scored above the cut-off score 3 months after the surgery compared with the pre-operative percentage, the level of depressive complaints did not differ significantly from the pre-operative level at 3 months after the surgery (*Z* = 1.232, *p* = 0.218). As with the anxiety level, no significant difference was observed between the level of depressive complaints at 4 weeks and 3 months after the surgery (*Z* = 1.562, *p* = 0.118) (see Table 2).

### PTSD complaints

Four weeks after the surgery (T1), 3 patients (6.4%) had a score that was suggestive of PTSD. After 3 months (T2), only 1 patient (2.0%) met this criterion (see Fig. 1). This patient had also reported complaints that were suggestive of PTSD at 4 weeks after the surgery. The average total SRIP score was below the cut-off score of 52 points at both measurements, see Table 2. The total scores of PTSD complaints were, both at T1 and T2 not associated with sex or age (see Online Resource 1).

Only a small percentage of the patients scored above the cut-off on the SRIP. To still be able to make statements regarding the events to which the PTSD complaints were attributed, the VAS scores were examined for the patients who reported *any* PTSD complaints (total score higher than the minimum score of 22). 4 weeks after the surgery, this applied to 46 (98%) of the 47 patients who had completed the SRIP at this measurement. Of these patients, 27.9% attributed the PTSD complaints mostly to the discovery of the tumour, 23.3% attributed them to the surgery, and 41.9% attributed them to the post-operative neuropathological diagnosis. 3 months after the surgery, 44 (80%) of the 50 patients who had completed the SRIP at this measurement reported PTSD complaints to some extent. Of these patients, 42.1% attributed the complaints mostly to the discovery of the tumour, 21.1% attributed them to the surgery, and 34.2% attributed them to the postoperative neuropathological diagnosis.

## Discussion

The aim of the current study was to examine to what extent psychological complaints increase after awake craniotomy in patients with brain tumours. This study shows that the number of patients that show post-operatively a deterioration in depressive- and anxiety symptoms compared to pre-operative levels of these symptoms, is comparable to the number of patients who show an improvement in these symptoms after the surgery. On group-level, there are no indications that awake craniotomy leads to more anxiety or depressive complaints compared with the pre-operative level of complaints. Moreover, it is concluded that post-traumatic stress disorders after awake craniotomy are very rare. However, some post-traumatic stress complaints were reported to a lesser extent. It is noteworthy that these complaints were least attributed to the surgery itself; the complaints were attributed more to the discovery of the tumour and the postoperative neuropathological diagnosis, both in the short term and in the longer term after surgery.

The results of this study correspond with the findings of previous studies. For example, Hejrati and colleagues (2019) [15] found no difference in the degree of anxiety, depressive complaints and post-traumatic stress before and after awake craniotomy. Although the procedure used in their study (awake-awake-asleep) is not entirely the same as the procedure used in current study, the patients were also conscious until the tumour resection was completed. In addition, a study of Bakhshi et al. (2021) concludes that after awake craniotomy there is no additional incidence of postoperative depression compared with the resection of brain tumours under general anesthesia [3]. A recent systematic review from Mofatteh and colleagues showed that in more than 95% of the included studies in their review awake craniotomy did not lead to increased level of stress, anxiety or depression [21]. Regarding PTSD symptoms, a study by Milian et al. (2013) showed that 12.5% of the patients displayed symptoms of PTSD following an awake-awake-awake craniotomy procedure [19]. However, a review by the same researchers showed that patients do not develop an actual post-traumatic stress disorder following an awake craniotomy [20]. Recent research by Starowicz-Filip et al. (2022) found no indications for clearly higher scores on questionnaires used for the detection of PTSD complaints [24]. This is in line with the findings of the current study; only 6% of the patients reported a level of complaints that was suggestive of PTSD 4 weeks after the surgery, and that level of complaints was only present in 2% (1/50) of the patients 3 months after the surgery. It is noteworthy that this is a clearly lower percentage than those known for other types of surgeries. For example, El-Gabalawy et al. (2019) describe that an average of 20% of the patients experience clinically significant post-traumatic stress after a surgical procedure, with a range of 16% to 51% for specific types of surgeries, such as lung resections or vascular abdominal surgeries [12]. The difference in PTSD symptoms between these surgeries and an awake craniotomy may be explained by the fact that patients who undergo an awake craniotomy receive intensive psychological support. Finally, our results align with the study of Leon-Rojas et al., in which patients were asked about their experience during an awake craniotomy. More than 90% of the patients reported willingness to repeat the procedure if medically necessary [17].

This study has formed a clear picture of the prevalence of psychological complaints after awake craniotomy over the short and longer term. Nevertheless, there are some limitations that deserve mention. The first limitation is missing data on the post-operative measurements. This may have resulted in a distorted image, for example because patients without complaints might be less likely to complete the questionnaire. On the other hand, it is also possible that patients with severe psychological complaints did not complete the questionnaire because they did not want to be confronted with their complaints. Another limitation is that the level of anxiety, depressive complaints and post-traumatic stress was measured by means of self-reporting. This brings the risk of over-reporting or under-reporting. As a result, only statements can be made regarding the subjective complaints that patients experience and report; it does not provide an in-depth evaluation of potential psychiatric disorders. Another limitation of this study is that it did not examine factors that might predict the intra-individual changes (improvement or deterioration) in depressive and anxiety symptoms that were found in a subgroup of the patients. One factor that could play a role in the course of psychological complaints after surgery, is the use of adjuvant tumour therapy after the surgery. Previous research has shown that 46% of cancer patients who undergo radiotherapy display some form of psychopathology at some point during their treatment, such as an anxiety disorder, an adjustment disorder or depression [4]. As some of the brain tumour patients also undergo another form of treatment in addition to surgery, it is recommended that the influence of this treatment be considered in future research. Other factors related to the surgery itself, such as any complications or loss of function during the procedure, might also influence the course of psychological complaints. In addition, the presence of pre-morbid psychological disorders should be considered as a possible determinant of changes in psychological complaints after surgery. A history of psychopathology prior to the tumour diagnosis (e.g. early psychological trauma, anxiety disorder or depressive disorder) could make someone more vulnerable for developing psychological complaints later in life [7, 22]. Moreover, personal factors such as coping, personality and the amount of experienced social support may influence the degree to which patients develop psychological complaints. A final limitation of this study is that the psychological complaints were not monitored for longer than 3 months after the surgery. This study emphasized the influence of the surgery on the patient’s psychological complaints. Based on the data, we can make no statements regarding the level of psychological complaints over the longer term. The review by El-Gabalawy et al. (2019) indicates that in the first year after surgery the course of post-traumatic stress complaints can vary: although some studies have shown that the number of patients with PTSD increases others show a decrease, a stable pattern, or fluctuations in post-traumatic stress after surgery [12]. To gain more insight in the course of depressive and anxiety symptoms over the long-term post-surgery, longitudinal studies with extended follow up periods are recommended.

## Conclusion

This study presents a valuable addition to the existing literature on the psychological impact of awake craniotomies because of the relatively large study sample and its longitudinal design. Although some of the patients reported anxiety and depressive complaints after undergoing awake craniotomy, based on group level there was no indication for an increase in these complaints compared to the pre-operative situation. Symptoms of post-traumatic stress are present in a limited degree after awake craniotomy, but the level of these complaints is very rarely suggestive of a PTSD. Nevertheless, health care professionals involved in awake craniotomy should be aware of possible psychological complaints in each individual patient and should provide psychological support when needed.

## Supplementary information

Below is the link to the electronic supplementary material.

Supplementary file1 (PDF 97 KB)
