# Supplementary material for: Awake craniotomy does not lead to increased psychological complaints
Source: Acta Neurochir (Wien). 2023 May 24;165(9):2505–12. doi: 10.1007/s00701-023-05615-5 (PMC10477129; doi:10.1007/s00701-023-05615-5)
Supplement: Supplementary file 1 — Supplementary file1 (PDF 97 KB) [file 701_2023_5615_MOESM1_ESM.pdf]

**ONLINE RESOURCE 1 -** Supplementary information

**Title article:** Awake craniotomy does not lead to increased psychological complaints

**Journal:** Acta neurochirurgica

**Authors:** I.M.C. Huenges Wajer[1,2], PhD – J. Kal[1,2], MSc – P.A.J.T. Robe[1], MD, PhD – M. J. E. van Zandvoort[1,2], PhD – C. Ruis[1,2], PhD

[1] *Department of Neurology and Neurosurgery, Rudolf Magnus Institute for Neuroscience, University Medical Center Utrecht, Utrecht, The Netherlands*

[2] *Experimental Psychology, Helmholtz Institute, Utrecht University, Utrecht, The Netherlands*

**Corresponding author:** I.M.C. Huenges Wajer (I.M.C.HuengesWajer@umcutrecht.nl)

Table 1: Kendall’s tau correlation between sex, age and complaints of anxiety, depression and PTSD

| | Anxiety | | | | | | Depression | | | | | | PTSD | | | |
|---|---|---|---|---|---|---|---|---|---|---|---|---|---|---|---|---|
| | T0 | | T1 | | T2 | | T0 | | T1 | | T2 | | T1 | | T2 | |
| | $r_\tau$ | $p$ | $r_\tau$ | $p$ | $r_\tau$ | $p$ | $r_\tau$ | $p$ | $r_\tau$ | $p$ | $r_\tau$ | $p$ | $r_\tau$ | $p$ | $r_\tau$ | $p$ |
| Sex | .321 | .012* | .210 | .092 | .194 | .109 | .327 | .011* | .185 | .141 | .124 | .306 | .131 | .292 | .048 | .689 |
| Age | -.144 | .180 | -.066 | .530 | .007 | .948 | -.008 | .939 | .091 | .388 | .197 | .053 | .016 | .876 | -.054 | .591 |

*T0 = pre-operative; T1 = 4 weeks after surgery; T2 = 3 months after surgery*

*$p<.05$
